# Airway Management in Obstructive Sleep Apnea: A Comprehensive Review of Assessment Strategies, Techniques, and Technological Advances

**DOI:** 10.3390/healthcare13151823

**Published:** 2025-07-26

**Authors:** Mario Giuseppe Bellizzi, Annalisa Pace, Giannicola Iannella, Antonino Maniaci, Daniele Salvatore Paternò, Simona Tutino, Massimiliano Sorbello, Salvatore Maria Ronsivalle, Giuseppe Magliulo, Antonio Greco, Armando De Virgilio, Patrizia Mancini, Enrica Croce, Giulia Molinari, Daniela Lucidi, Jerome R. Lechien, Antonio Moffa, Alberto Caranti, Luigi La Via

**Affiliations:** 1 Department of ‘Organi di Senso’, University “Sapienza”, Viale dell’Università 33, 00185 Rome, Italy; mariobellizzi1993@gmail.com (M.G.B.); annalisa.pace@uniroma1.it (A.P.); giuseppe.magliulo@uniroma1.it (G.M.); antonio.greco@uniroma1.it (A.G.); armando.devirgilio@uniroma1.it (A.D.V.); p.mancini@uniroma1.it (P.M.); enrica.croce@uniroma1.it (E.C.); 2 Faculty of Medicine and Surgery, University of Enna “Kore”, 94100 Enna, Italy; tnmaniaci29@gmail.com (A.M.); massimiliano.sorbello@gmail.com (M.S.); salvatore.ronsivalle@aspct.it (S.M.R.); 3 Department of Anaesthesia and Intensive Care, Giovanni Paolo II Hospital, 97100 Ragusa, Italy; paternomd@icloud.com (D.S.P.); simona.tutino@studenti.unicz.it (S.T.); 4 Otolaryngology and Audiology Unit, IRCCS Azienda Ospedaliero-Universitaria Policlinico di Sant’Orsola, 40138 Bologna, Italy; giulia.molinari8@unibo.it; 5 Department of Experimental, Diagnostic and Specialty Medicine—DIMES, Alma Mater Studiorum University, 40126 Bologna, Italy; 6 Department of Otolaryngology, Ospedale Santa Maria delle Croci di Ravenna—Bologna University, 48121 Ravenna, Italy; daniela.lucidi@unibo.it; 7 Department of Human Anatomy and Experimental Oncology, Faculty of Medicine, UMONS Research Institute for Health Sciences and Technology, University of Mons (UMons), 7000 Mons, Belgium; jerome.lechien@umons.ac.be; 8 Integrated Therapies in Otolaryngology, Fondazione Policlinico Universitario Campus Bio-Medico, 00128 Rome, Italy; a.moffa@policlinicocampus.it; 9 GVM Care & Research ENT Consultant, San Pier Damiano Hospital, University of Ferrara, Via Portisano 1, 48018 Faenza, Italy; dott.albertocaranti@gmail.com; 10 Department of Anesthesia and Intensive Care, University Hospital Policlinico “G. Rodolico—San Marco”, 24046 Catania, Italy; luigilavia7@gmail.com

**Keywords:** obstructive sleep apnea, difficult airway management, videolaryngoscopy, flexible endoscopic intubation, airway assessment, apneic oxygenation, hybrid airway devices, preoxygenation, anesthesia guidelines, perioperative complications

## Abstract

**Background**: Airway management in patients with obstructive sleep apnea (OSA) presents unique challenges for anesthesiologists and other airway practitioners. This comprehensive review examines current evidence and clinical practices for managing difficult airways in this high-risk population. OSA is characterized by specific anatomical and physiological alterations that increase both the likelihood of encountering difficult intubation and the risk of rapid desaturation during airway manipulation. **Methods**: Preoperative assessment of OSA patients requires integration of traditional difficult airway evaluation with OSA-specific considerations, including severity indices, oxygen desaturation patterns, and continuous positive airway pressure dependency. Conventional direct laryngoscopy often proves inadequate in these patients, prompting the development and refinement of alternative approaches. Videolaryngoscopy has emerged as a particularly valuable technique in OSA patients, offering improved glottic visualization while maintaining physiologic positioning. Flexible endoscopic techniques, particularly awake flexible bronchoscopic intubation, remain essential for high-risk scenarios, though they require considerable expertise. **Results**: Recent technological innovations have produced hybrid devices combining multiple modalities to address the specific challenges presented by OSA patients. Adjunctive tools and techniques, including specialized introducers, exchange catheters, and high-flow nasal oxygen, play critical roles in extending safe apnea time and facilitating successful intubation. Professional society guidelines now incorporate OSA-specific recommendations, emphasizing thorough preparation, appropriate device selection, and comprehensive monitoring. **Conclusions**: Effective management ultimately requires not only appropriate technology but also systematic preparation, strategic device selection, and meticulous execution. As OSA prevalence continues to rise globally, optimizing airway management approaches for this challenging population remains a critical priority for patient safety.

## 1. Introduction

Obstructive sleep apnea (OSA) represents one of the most prevalent sleep-related breathing disorders, affecting approximately 10–30% of the adult population worldwide [1]. This condition is characterized by repetitive episodes of partial or complete upper airway collapse during sleep, resulting in oxygen desaturation, sleep fragmentation, and significant cardiovascular and metabolic consequences [2]. Obesity is the major contributing factor for OSA, and the prevalence of OSA continues to rise alongside the global obesity epidemic, with recent epidemiological studies suggesting that moderate to severe OSA affects 23.4% of women and 49.7% of men aged 40–85 years [3]. Other important factors contributing to OSA are age, male gender, family history, smoking, alcohol consumption, and oxidative stress [4].

**Figure 1:**
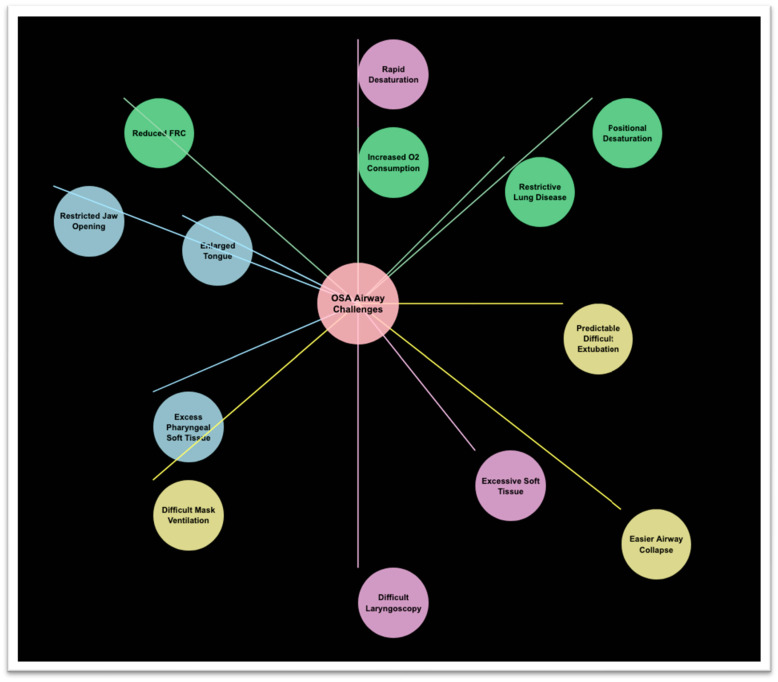
Preoperative airway assessment in OSA patients: Key challenges and considerations. This figure illustrates the essential components of preoperative airway evaluation specific to OSA patients, highlighting anatomical factors, physiological alterations, and risk assessment tools that influence airway management decisions.

From an anesthesiologic perspective, OSA presents unique challenges during perioperative airway management, particularly during orotracheal intubation and post-extubation period (Figure 1). The anatomical and physiological alterations associated with OSA significantly increase the risk of difficult mask ventilation, challenging laryngoscopy, and potentially life-threatening complications during intubation attempts and in the immediate postoperative course [5]. Recent data from the Multicenter Perioperative Outcomes Group database demonstrated that OSA patients have a 2.3-fold increased risk of difficult mask ventilation and a 1.8-fold increased risk of difficult intubation compared to non-OSA patients [6], which may further increase in case of unplanned post-procedural reintubation.

The pathophysiological factors contributing to these challenges are multifaceted. OSA patients typically exhibit a constellation of unfavorable airway characteristics, including reduced pharyngeal dimensions, excess pharyngeal soft tissue, macroglossia, and a shortened thyromental distance [7]. Additionally, neck circumference exceeding 43 cm in men and 41 cm in women strongly correlates with both OSA severity and difficult airway management [8,9]. The presence of retrognathia and a high Mallampati score further compounds these difficulties, creating scenarios where conventional intubation approaches may prove inadequate [10].

Beyond anatomical considerations, physiological factors also contribute to the complexity of airway management in this population. OSA patients demonstrate increased sensitivity to the respiratory-depressant effects of sedatives and opioids, leading to a more rapid onset of airway collapse and oxygen desaturation during induction of anesthesia [11]. Recent studies have documented that OSA patients desaturate three times faster than their non-OSA counterparts during apneic periods [12]. The pathophysiological reason for this sensitivity comprehends the reduced functional residual capacity, together with an increased oxygen consumption and ventilation-perfusion mismatch. This reduced oxygen reserve, coupled with an increased oxygen consumption due to the higher work of breathing, creates a narrow window for successful intubation before critical desaturation occurs [13].

Furthermore, OSA patients often present with comorbidities that further complicate airway management, including obesity, hypertension, coronary artery disease, and type 2 diabetes [14]. A retrospective analysis of 45,000 surgical procedures revealed that OSA patients experienced a 44% higher rate of unexpected post-induction airway management difficulties compared to matched non-OSA controls [15].

The expansion of ambulatory and office-based procedures has placed additional emphasis on the need for reliable and efficient airway management strategies for OSA patients, as these settings may have limited resources for managing unanticipated difficult airways [16]. The introduction of novel intubation devices and techniques has therefore become increasingly important in mitigating these risks.

This narrative review aims to comprehensively examine recent advancements in orotracheal intubation devices specifically relevant to the OSA population. By exploring innovations across videolaryngoscopy, flexible endoscopic systems, hybrid devices, and adjunctive tools, we seek to provide clinicians with an evidence-based framework for selecting appropriate airway management strategies for this challenging patient population. The review will also identify gaps in current knowledge and highlight promising directions for future research and development.

## 2. Search Strategy

This comprehensive narrative review was conducted to evaluate the current evidence and clinical practice concerning airway management techniques specifically tailored for patients with OSA. We performed a thorough literature search of major electronic databases, including PubMed/MEDLINE, Embase, Cochrane Library, and Web of Science. The search strategy encompassed publications from January 2000 through October 2024 to ensure inclusion of both established practices and emerging technologies.

Search terms included various combinations of keywords related to airway management (“intubation,” “airway management,” “difficult airway,” “videolaryngoscopy,” “fiberoptic intubation”) paired with terms describing the patient population (“obstructive sleep apnea,” “OSA,” “sleep-disordered breathing,” “obesity hypoventilation syndrome”). Additional terms targeting specific devices and techniques were also incorporated to ensure comprehensive coverage of the topic.

We prioritized inclusion of randomized controlled trials, prospective observational studies, meta-analyses, and systematic reviews addressing airway management in adult patients with confirmed or suspected OSA. Retrospective studies, case series, and case reports were included when they provided unique insights into device performance or technique application specifically relevant to OSA patients. We also searched for data corresponding to international guidelines and statements issued by learned societies, including those published on official websites. Articles focusing exclusively on pediatric populations were excluded, as were those addressing surgical management of OSA without discussion of perioperative airway considerations.

Current practice guidelines and consensus statements from major anesthesiology and airway management societies worldwide were reviewed to establish the standard of care across different practice environments. These included but were not limited to the American Society of Anesthesiologists, Difficult Airway Society (UK), European Society of Anaesthesiology, Society of Airway Management, and the Society of Anesthesia and Sleep Medicine.

Each identified publication was evaluated for methodological quality, relevance to clinical practice, and specific applicability to the OSA population. Priority was given to studies that specifically addressed the anatomical and physiological challenges unique to OSA patients rather than those that merely included OSA as one of many comorbidities in a broader difficult airway population.

The extracted data was organized thematically to address the core aspects of airway management in OSA patients: preoperative assessment, conventional device limitations, videolaryngoscopy applications, flexible endoscopic techniques, hybrid technologies, adjunctive tools, and guideline implementation. Within each thematic area, evidence was synthesized to identify consistent findings, areas of controversy, and gaps in current knowledge.

Particular attention was paid to comparative studies evaluating different devices or techniques specifically in OSA patients, as these provide the most direct evidence for clinical decision-making in this population. Where direct comparative evidence was lacking, we synthesized findings from multiple sources to develop reasoned conclusions about optimal approaches.

This comprehensive narrative approach allowed for integration of diverse evidence sources to provide clinically relevant guidance while acknowledging the ongoing evolution of the field. The resulting review aims to provide clinicians with a thorough understanding of current best practices and emerging technologies for airway management in this challenging patient population.

## 3. Preoperative Airway Assessment in OSA Patients

Accurate preoperative airway assessment remains the cornerstone of safe anesthetic management for any patient, and even more so for OSA patients. A systematic and comprehensive evaluation allows clinicians to anticipate difficulties, formulate contingency plans, and select appropriate intubation devices [17]. Traditional airway assessment tools have demonstrated variable performance in predicting intubation challenges specifically in the OSA population. The Mallampati classification, which evaluates visibility of oropharyngeal structures, has shown modest sensitivity (71%) but limited specificity (56%) for predicting difficult intubation in OSA patients according to a prospective cohort study by Naguib et al. [18]. When combined with additional metrics, such as thyromental distance, sternomental distance, and neck circumference in a multivariate model, predictive accuracy improves significantly (area under ROC curve 0.79 vs. 0.67 for Mallampati alone) [19]. The upper lip bite test remains the most reliable test to predict difficult laryngoscopy [20], but poor or no data are available for OSA sub-populations.

The STOP-BANG questionnaire, originally developed as a screening tool for OSA, has emerged as a valuable predictor of difficult airway management. This eight-point assessment evaluates Snoring, Tiredness, observed apneas, blood Pressure, BMI, Age, Neck circumference, and Gender. A recent meta-analysis by Singh et al. found that STOP-BANG scores ≥ 5 were associated with a 4.3-fold increased risk of difficult intubation (95% CI: 2.8–6.6) [21]. The integration of STOP-BANG into preoperative protocols has been shown to reduce unanticipated difficult airway scenarios [22,23].

Modified neck circumference, calculated as neck circumference plus specific adjustments for OSA risk factors, provides another targeted assessment approach, demonstrating that a modified neck circumference exceeding 50 cm predicted difficult laryngoscopy with 78% sensitivity and 82% specificity, outperforming standard metrics in OSA patients [24]. Similarly, the upper lip bite test has shown promising results in this population, with Grade III performance (inability to bite upper lip) carrying a 5.6-fold increased risk of Cormack–Lehane grade 3–4 views during direct laryngoscopy [20,25].

Ultrasound-based airway assessment has gained traction as a non-invasive, quantifiable method for predicting difficult intubation in OSA patients. Measurement of anterior neck soft tissue thickness at the level of the hyoid bone and thyrohyoid membrane correlates significantly with intubation difficulty [26]. Recent evidence found that ultrasound measurement of soft tissue thickness at hyoid bone, thyrohyoid membrane, and anterior commissure of vocal cord are good independent predictors for difficult laryngoscopy [27,28,29].

Drug-induced sleep endoscopy (DISE) provides dynamic visualization of upper airway collapse during simulated sleep conditions. Although traditionally used for surgical planning in OSA treatment, emerging evidence suggests that DISE findings correlate with difficult intubation risk. Ravesloot et al. identified that complete concentric collapse at the velum during DISE was associated with a 3.1-fold increased risk of difficult laryngoscopy in subsequent procedures under general anesthesia [30]. The VOTE classification (Velum, Oropharynx, Tongue base, Epiglottis) obtained during DISE offers additional insights into the specific anatomical sites contributing to airway obstruction, potentially guiding intubation device selection [31].

Integration of multiple assessment modalities into composite scores improves predictive accuracy. The El-Ganzouri Risk Index, which combines seven airway parameters, demonstrated superior performance in OSA patients compared to individual tests, with 93% sensitivity and 85% specificity for difficult intubation when using a modified threshold of ≥5 points [32]. Similarly, the combined Wilson Risk Score and STOP-BANG assessment outperformed either tool alone in a prospective evaluation of 420 OSA patients undergoing elective surgery [33]. Nevertheless, both tests provide a quantitative risk assessment rather than a qualitative one, with unclear addressing of an airway management technique [34].

Despite these advancements, no single assessment tool achieves perfect predictive accuracy, highlighting the importance of employing multiple complementary evaluation methods. A comprehensive approach incorporating both static anatomical assessments and dynamic evaluations of airway function provides the most reliable prediction of intubation difficulties in OSA patients [35], which could without a doubt be included in the concept of the physiologically difficult airway [36]. Furthermore, preoperative identification of high-risk patients enables appropriate preparation, including equipment selection, personnel allocation, consideration of awake intubation techniques when indicated and pre-procedural assessment of post-procedural level of care [37].

## 4. Conventional Devices and Their Limitations in OSA

Conventional airway management tools, while widely available and familiar to practitioners, present significant limitations when applied to patients with OSA. Direct laryngoscopy (DL), the traditional gold standard for tracheal intubation, has shown reduced first-attempt success rates in OSA patients compared to their non-OSA counterparts. A retrospective analysis by Saasouh et al. demonstrated a 37% reduction in first-pass success with Macintosh blade DL in patients with severe OSA (Apnea–hypopnea index [AHI] > 30) [38]. The fundamental limitation of DL in this population stems from its requirement for alignment of the oral, pharyngeal, and laryngeal axes to achieve glottic visualization—a configuration frequently unattainable in OSA patients due to their altered airway anatomy [39].

Several factors contribute to the inadequacy of conventional Macintosh and Miller blades in the OSA population. The restricted oropharyngeal space, characteristic of OSA, limits blade insertion and positioning. In a comparative anatomical study using magnetic resonance imaging, Schwartz et al. documented an average 41% reduction in retropalatal airway diameter in OSA patients compared to matched controls, severely compromising the working space available for laryngoscope manipulation [40]. Furthermore, the increased posterior displacement of the tongue and epiglottis during anesthesia induction disproportionately affects OSA patients, with pharyngeal closing pressures increasing by an average of 11.8 cmH_2_O compared to 5.3 cmH_2_O in non-OSA subjects [41].

Modified laryngoscope blades designed to address these limitations have shown modest improvements but remain suboptimal. The McCoy levering laryngoscope improved Cormack–Lehane grade by at least one level in 63% of difficult airways in OSA patients, yet still resulted in a 22% failed intubation rate in this cohort [42]. Similarly, the Bullard laryngoscope, with its anatomically curved design, failed to significantly improve first-attempt success rates [43].

Supraglottic airway devices (SADs), including various generations of laryngeal mask airways (LMAs), present their own set of challenges in the OSA population. While often proposed as rescue devices during failed intubation, their effectiveness in establishing and maintaining a patent airway might be compromised in OSA patients. Ramachandran et al. reported a 4.4-fold increased risk of inadequate ventilation when using first-generation LMAs in OSA patients compared to matched controls [44]. The diminished pharyngeal space and excessive tissue create suboptimal seating of the SAD, leading to air leaks, inadequate ventilation, and potential airway obstruction [45].

Second-generation SADs incorporating gastric access and improved sealing mechanisms demonstrate better performance but may still encounter limitations in severe OSA. A prospective evaluation of the ProSeal LMA in 90 patients with severe OSA revealed a 28% incidence of significant air leaks requiring device repositioning or replacement, compared to just 7% in mild/moderate OSA [46]. Furthermore, oropharyngeal leak pressures were significantly lower in OSA patients (22.4 ± 4.7 cmH_2_O vs. 26.8 ± 3.9 cmH_2_O), potentially compromising ventilation when higher airway pressures are required [47].

Standard intubating stylets and bougies also demonstrate reduced utility in OSA patients. The rigid nature of these devices often fails to accommodate the exaggerated anterior curvature of the airway commonly observed in OSA. Wang et al. reported 73% first-attempt success rates with gum-elastic bougie-assisted intubation in non-OSA difficult airways, but only 44% in matched OSA patients with comparable Cormack–Lehane grades [48]. The limited maneuverability of conventional stylets in the confined oropharyngeal space of OSA patients further compromises their effectiveness [49].

Blind intubation techniques using devices such as the intubating LMA (Fastrach^®^—Teleflex Medical, Athlone, Ireland) show diminished success rates in the OSA population. A multicenter prospective study by Shiga et al. found first-attempt blind intubation success rates through the Fastrach LMA were significantly lower in OSA patients compared to non-OSA controls with predictors of difficult airways (67% vs. 88%) [50]. The altered relationship between the epiglottis, glottis, and surrounding structures in OSA contributes to this reduced performance [51].

Conventional optical stylets like the Bonfils^®^ intubation fiberscope (Karl Storz, Tuttlingen, Germany), while providing better visualization than blind techniques, still encounter limitations in severe OSA. Restricted jaw mobility and reduced submandibular compliance restrict device manipulation, with a prospective comparison finding significantly longer intubation times in OSA patients compared to matched controls (47 s vs. 29 s, *p* < 0.001) [52].

Emergency rescue techniques including cricothyrotomy are further complicated in OSA patients by difficult neck anatomy, including increased adipose tissue and altered landmarks [53]. An observational study by Lavelle et al. demonstrated the possibility of ultrasonographic identification of the cricothyroid membrane [54]. This anatomical challenge underscores the importance of preventing “cannot intubate, cannot oxygenate” scenarios through appropriate initial device selection in this high-risk population [55].

The limitations of conventional airway management tools in OSA patients have driven the development and adoption of advanced technologies specifically addressing these anatomical and physiological challenges, as will be discussed in subsequent sections of this review.

## 5. Innovations in Videolaryngoscopy

Videolaryngoscopy (VL) represents a significant advancement in airway management technology, with potential for supplementary benefit in OSA patients. By providing indirect glottic visualization through distal video cameras “looking around the corner”, these devices effectively circumvent many of the anatomical challenges that compromise conventional direct laryngoscopy. Modern videolaryngoscopes have evolved into various designs and configurations, each offering distinct features with different potential advantages for the management of OSA-associated difficult airways (Table 1).

The GlideScope^®^ system (Verathon Medical, bothell, Washington, DC, USA), with its distinctive 60-degree angulated blade, has demonstrated superior performance in OSA patients. In a landmark randomized controlled trial involving 200 patients with severe OSA (AHI > 30), Andersen et al. reported significantly higher first-attempt success rates with the GlideScope^®^ compared to direct laryngoscopy (91% vs. 68%, *p* < 0.001) [56]. The improved glottic visualization was particularly evident in patients with high Mallampati scores and limited neck mobility. A subsequent meta-analysis by Healy et al. confirmed these findings, demonstrating that videolaryngoscopy reduced failed intubations in OSA patients by 76% compared to conventional approaches (RR 0.24, 95% CI 0.12–0.49) [57].

The McGrath^®^ MAC videolaryngoscope (Medtronic, Dublin, Ireland) employs either a more traditional Macintosh-shaped or a hyperangulated blade, both equipped with CCD sensors, making it particularly suitable for clinicians transitioning from direct laryngoscopy. Aziz et al. conducted a prospective observational study specifically examining the McGrath^®^ in 167 OSA patients with predicted difficult airways, finding a 94% first-attempt success rate and average intubation time of 38 s [58]. The device’s familiar blade shape combined with improved visualization appears to reduce the learning curve for practitioners while maintaining high success rates in challenging OSA airways.

The C-MAC^®^ system (Karl Storz, Tuttlingen, Germany) offers versatility through interchangeable blades, including the hyperangulated D-blade^®^ specifically designed for difficult airways. In a comparative study of different videolaryngoscopes in OSA patients, Cavus et al. found that the C-MAC^®^ D-blade provided superior glottic visualization (percentage of glottic opening score > 90%) in 86% of patients with severe OSA, compared to 72% with standard videolaryngoscopes and 48% with direct laryngoscopy [59]. The device’s design enables successful navigation of the exaggerated anterior airway curvature commonly found in OSA patients while maintaining sufficient space for endotracheal tube manipulation.

The Airtraq^®^ (Prodol, Biscay, Spain), a channeled optical laryngoscope, offers a guided approach to tube delivery that addresses the challenges of tube advancement often encountered in OSA patients. Maharaj et al. demonstrated that the Airtraq^®^ reduced intubation time by 41% and improved first-attempt success rates (96% vs. 72%) compared to direct laryngoscopy in OSA patients with Mallampati scores of III–IV [60]. The integration of an endotracheal tube channel provides a significant advantage in the limited oropharyngeal working space typical of OSA anatomy by eliminating the need for separate tube manipulation.

Comparative studies between different videolaryngoscope designs have yielded important insights regarding their relative efficacy in OSA-specific airways. Wasem et al. conducted a randomized crossover trial comparing four videolaryngoscopes (GlideScope^®^, C-MAC^®^, McGrath^®^, and King Vision^®^ (Ambu, Ballerup, Denmark)) in 88 patients with severe OSA, finding that hyperangulated designs (GlideScope^®^ and D-blade^®^) provided superior glottic visualization but slightly longer intubation times compared to more anatomically shaped blades [61]. This suggests that the optimal device selection may depend on the specific anatomical challenges and operator experience in individual OSA cases.

Video laryngoscopy has demonstrated particular benefits in managing OSA patients in emergency situations where the risks of difficult airway management are magnified. A multicenter registry analysis by Sakles et al. found that videolaryngoscopy reduced the incidence of failed intubation in emergency department patients with OSA by 67% compared to direct laryngoscopy (adjusted OR 0.33, 95% CI 0.24–0.46) [62]. This advantage was especially pronounced in patients with multiple difficult airway predictors, highlighting the value of these devices in high-stakes scenarios.

The combination of videolaryngoscopy with apneic oxygenation techniques has shown synergistic benefits in OSA patients. Wong et al. demonstrated that high-flow nasal oxygen during videolaryngoscopy-guided intubation significantly extended safe apnea time in OSA patients compared to conventional preoxygenation (6.4 min vs. 3.5 min until SpO_2_ < 90%) [63]. This extended safe apnea window is particularly valuable given the reduced functional residual capacity and increased oxygen consumption characteristic of OSA patients.

Beyond their intubation success rates, videolaryngoscopes offer substantial educational and quality improvement benefits. The shared view between operator and assistants enables real-time guidance and improved team coordination during challenging OSA intubations. Griesdale et al. documented that implementation of a videolaryngoscopy-based airway management program for OSA patients reduced unplanned ICU admissions due to airway complications by 56% over a two-year period [64].

Despite their advantages, current videolaryngoscopes still present certain limitations in OSA management. Blood or secretions can obscure camera views, anti-fogging measures occasionally fail, and some designs remain bulky for patients with restricted mouth opening. Additionally, while videolaryngoscopes improve glottic visualization, tube delivery can remain challenging in severe OSA anatomy, necessitating adjunct techniques such as stylet use or bougie guidance [65]. Emerging videolaryngoscope designs incorporating channels, articulating stylets, and suction capabilities aim to address these remaining limitations, potentially further improving outcomes in this challenging patient population [66].

## 6. Flexible Bronchoscopy Intubation and Awake Techniques

Flexible bronchoscopy intubation (FI), particularly in the awake patient, remains a cornerstone technique for managing high-risk airways in OSA patients. This approach provides several distinct advantages in this population, offering preservation of spontaneous ventilation, maintenance of pharyngeal muscle tone, and direct visualization of airway structures that may be distorted by OSA-related anatomical changes. For patients with severe OSA and multiple predictors of difficult intubation, awake FI is often considered the safest approach to securing the airway.

The decision to pursue awake intubation in OSA patients should be guided by a structured risk assessment. Ahmad et al. developed a specific decision algorithm for OSA patients that incorporates both standard difficult airway predictors and OSA-specific factors, such as AHI severity, nocturnal desaturation patterns, and continuous positive airway pressure (CPAP) dependency [67]. Application of this algorithm to 412 OSA patients scheduled for surgery resulted in appropriate selection of awake FI for high-risk cases, with no cases of failed intubation or significant desaturation events in the awake FI group [68].

Patient preparation is critical to successful awake FI, particularly in OSA patients. Traditional antisialagogues, such as glycopyrrolate, must be used judiciously, as Hillman et al. demonstrated that glycopyrrolate increases upper airway collapsibility in OSA patients by approximately 24% compared to controls [69]. This effect is likely due to increased mucosal dryness and reduced surface tension, which normally helps maintain airway patency. A modified premedication regimen for OSA patients undergoing awake FI typically includes reduced doses of antisialagogues combined with nasal vasoconstrictors to optimize visualization while minimizing airway collapsibility [70].

Topical anesthesia techniques have evolved to provide more effective airway anesthesia while minimizing sedation requirements in OSA patients. The “spray-as-you-go” technique, where lidocaine is applied through the working channel of the bronchoscope, has demonstrated particular efficacy in OSA patients. Dabbagh et al. reported a 94% successful awake FI rate using this technique with minimal supplemental sedation in 75 patients with severe OSA [71]. Alternative approaches include nebulized lidocaine (2–4%), dedicated malleable atomizers and transcricoid injection of lidocaine, which provides effective glottic and subglottic anesthesia while minimizing the risk of lidocaine toxicity; this is an important consideration in OSA patients who often have reduced drug clearance due to associated comorbidities [72]. Specific topicalization techniques are widely described in dedicated guidelines [68].

Sedation during awake FI always requires careful titration; this is specifically true in OSA patients, as these individuals demonstrate heightened sensitivity to the respiratory-depressant effects of sedatives and opioids [73]. Dexmedetomidine has emerged as a preferred sedative agent for this population, providing anxiolysis and cooperation without significant respiratory depression. A randomized controlled trial by Chu et al. compared dexmedetomidine to remifentanil for awake FI in 60 patients with severe OSA, finding significantly higher minimum oxygen saturations (94% vs. 88%, *p* < 0.001) and lower incidence of apnea episodes (7% vs. 40%) in the dexmedetomidine group [74]. The preserved respiratory drive and maintained pharyngeal tone with dexmedetomidine make it particularly valuable for high-risk OSA patients.

Remifentanil has also been used effectively in low-dose, target-controlled infusions for awake FI in OSA patients. Vennila et al. demonstrated successful awake FI in 45 patients with severe OSA using target-controlled remifentanil infusions at 0.5–1.5 ng/mL, with high patient satisfaction and minimal respiratory depression [75]. The short half-life and rapid titratability of remifentanil allow for precise management of sedation levels during the procedure, although continuous monitoring of respiratory parameters remains essential.

Technical aspects of FI in OSA patients present distinct challenges compared to the general population. The altered upper airway anatomy, including enlarged tonsils, redundant pharyngeal tissue, and retrognathia, can complicate bronchoscope navigation. Johnson et al. conducted a radiographic comparison of awake FI in OSA versus non-OSA patients, documenting a significantly larger anterior displacement force required to navigate the characteristic anterior airway curve in OSA patients (1.2 N vs. 0.7 N, *p* < 0.01) [76]. This finding highlights the importance of using appropriate scope angulation techniques and considering more flexible ultrathin bronchoscopes for severe OSA cases.

Navigational strategies specific to OSA anatomy have been developed to improve FI success rates. The “jaw-thrust first” technique described by Durga et al. significantly improved the first-pass success rate of awake FI in OSA patients from 78% to 93% by creating maximal pharyngeal space before scope insertion [77]. Similarly, extension of the atlanto-occipital joint rather than the traditional “sniffing position” has been shown to optimize the pharyngeal space specifically in OSA patients, with MRI studies confirming an average 27% increase in retroglossal cross-sectional area with this modification [78].

Various adjunctive devices have been developed to facilitate FI in challenging OSA airways. The Berman airway and Williams airway support the tongue anteriorly and create a channel for bronchoscope advancement. A comparative study by Evans et al. found that the Williams airway provided superior bronchoscopic conditions in OSA patients compared to other oral airways, with improved visualization scores and shorter time to glottic identification (44 s vs. 67 s, *p* < 0.05) [79]. Specialized masks and endoscopic face masks that allow oxygen delivery during the procedure while providing access for the bronchoscope are particularly valuable in OSA patients who desaturate rapidly during apneic periods [80].

The combination of flexible bronchoscopy with videolaryngoscopy (channeled video-assisted fiberoptic intubation) has shown particular promise in managing complex OSA airways. Moore et al. reported a 99% success rate with this hybrid technique in 108 patients with severe OSA and multiple predictors of difficult intubation [81]. By providing simultaneous anterior airway displacement through the videolaryngoscope while navigating with the flexible bronchoscope, this approach addresses the specific anatomical challenges presented by OSA patients.

Despite its advantages, awake FI in OSA patients is not without limitations. Operator’s experience significantly impacts success rates [82], with novice practitioners demonstrating longer intubation times and higher failure rates specifically in OSA patients [83]. Additionally, patient anxiety and cooperation can present challenges, although modern pharmacological approaches and careful psychological preparation have improved tolerability. As technology advances, the integration of flexible endoscopy with other visualization techniques continues to evolve, further enhancing the management of this challenging patient population [84].

Interesting options come from awake intubation using videolaryngoscopes, which may combine a more familiar and user-friendly technique for anesthesiologists if compared to flexible bronchoscopy and the benefits of maintaining spontaneous breathing [68]. To date, no specific studies exploring the role of awake videolaryngoscopy for OSA patients have been published, but available data suggest a potential important role [85].

## 7. Hybrid Devices and Other Technological Advancements

**Figure 2:**
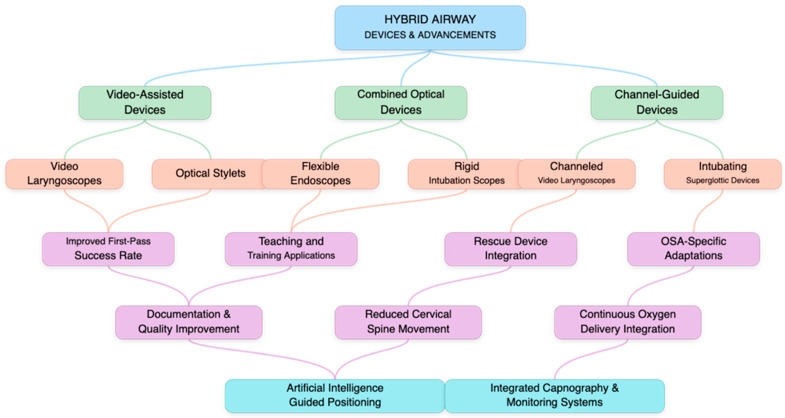
New perspectives and future challenges for technological advancements. This figure depicts emerging airway management technologies for OSA patients, including hybrid devices, video-assisted techniques, and advanced visualization systems, alongside future challenges and development opportunities in this rapidly evolving field.

The evolution of airway management devices has recently accelerated with the emergence of hybrid technologies that combine features from multiple device categories to address the specific challenges presented by OSA patients (Figure 2). These innovative tools integrate optical capabilities, video technology, and enhanced maneuverability to overcome the anatomical and physiological barriers encountered in this challenging population.

Video stylets represent a significant advancement in this hybrid category, combining the maneuverability of traditional stylets with the visualization capabilities of video technology. The Bonfils^®^ Retromolar Intubation Fiberscope (Karl Storz, Tuttlingen, Germany) has demonstrated particular utility in OSA patients with limited mouth opening. In a prospective study specifically targeting OSA patients with cervical spine limitations, Rudolph et al. reported a 93% first-attempt success rate with the Bonfils, compared to 58% with conventional direct laryngoscopy [86]. The rigid yet curved design allows navigation of the anterior airway curve characteristic of OSA while maintaining a slim profile suitable for restricted mouth opening.

Recent video stylet innovations include the Clarus Video System (CVS) (Clarus Medical, Plymouth, MN, USA), which incorporates an LED light source and wide-angle camera into a malleable intubating stylet. Maassen et al. evaluated this device in 64 OSA patients with predicted difficult airways, documenting a 95.3% success rate with mean intubation time of 26 s [87]. The ability to dynamically adjust the stylet shape during the procedure proved particularly valuable in adapting to the variable upper airway anatomy encountered in OSA patients.

The SensaScope^®^ (Acutronic MS, Hirzel, Switzerland), a hybrid S-shaped semi-rigid video stylet, offers another approach to OSA airways. Biro et al. conducted a comparative trial between the SensaScope^®^ and GlideScope^®^ in 80 patients with severe OSA, finding comparable success rates (92.5% vs. 95%) but significantly shorter average intubation times with the SensaScope^®^ (33 s vs. 47 s, *p* < 0.01) [88]. The device’s unique S-shaped design accommodates the anterior displacement of laryngeal structures in OSA while maintaining sufficient rigidity for tube delivery.

Optical-guided intubation tubes represent another hybrid approach garnering attention for OSA airway management. The Vivasight SL (ETView LTD—Ambu, Ballerup, Denmark), a single-lumen endotracheal tube with an integrated high-resolution camera, provides continuous visualization throughout the intubation process and subsequent tube positioning. Huitink et al. reported on its use in 120 patients with difficult airways, including 48 with confirmed OSA, achieving a 97.9% first-attempt success rate in the OSA subgroup [89]. The continuous visualization enabled real-time adjustments during tube advancement, addressing a common challenge in OSA patients, in which visualization of the glottis does not guarantee successful tube passage.

Channel-guided videolaryngoscopes represent a hybrid between standard videolaryngoscopes and optical stylets. The King Vision^®^ (Ambu, Ballerup, Denmark) with channeled blade exemplifies this approach, with Kleine-Brueggeney et al. demonstrating a 91% first-attempt success rate in 67 OSA patients with multiple predictors of difficult intubation [90]. The guided channel addresses the challenge of navigating the endotracheal tube through the complex airway curvature typical of OSA, while the video component provides continuous visualization throughout the procedure.

Three-dimensional visualization technology has emerged as a promising addition to airway management devices for OSA patients. The 3D McGrath VL (Medtronic, Dublin, Ireland) incorporates stereoscopic cameras that provide depth perception, addressing a significant limitation of traditional two-dimensional video systems. The enhanced depth perception proved particularly valuable for navigating the complex three-dimensional anatomy characteristic of OSA airways.

Independently on newer models, Videolaryngoscopes probably remain the best available tool for difficult laryngoscopy, hence remaining the first-choice devices also for OSA patients, as from the results of a recent meta-analysis carried out by Hansel et al. including 222 studies (219 RCTs, three quasi-RCTs) with 26,149 participants undergoing tracheal intubation, demonstrating that VLs of all designs likely reduce rates of failed intubation and result in higher rates of successful intubation on the first attempt with improved glottic views [91].

Magnetic guidance systems represent an innovative approach to facilitating tube delivery in challenging airways. The Magnetrac system utilizes magnetic tracking to guide endotracheal tube placement after glottic visualization has been achieved [92]. This technology addresses the frequent challenge in OSA patients, in which laryngeal visualization can be achieved, but endotracheal tube advancement remains difficult due to anterior angulation.

Robotic intubation systems have begun to emerge as the next frontier in complex airway management. The Kepler Intubation System (KIS) combines robotically controlled articulating stylet technology with high-definition visualization. In a preliminary study involving 25 patients with severe OSA and predicted difficult intubation, Hemmerling et al. reported a 92% first-attempt success rate with the KIS [93]. The precise control afforded by robotic articulation enables navigation through the complex airway anatomy encountered in OSA, while the operator remains physically distant from the patient—a potential advantage in scenarios requiring distancing or protection.

Augmented reality (AR) systems are being integrated into airway management to enhance operator guidance during difficult intubations. Intubation Difficulty with King Vision^®^ (Ambu, Ballerup, Denmark) and Truview Videolaryngoscope (Truphatek International Ltd., Netanya, Israel) in Manikin demonstrated that King Vision^®^ videolaryngoscope results in shorter intubation time and better glottic view [94]. Though still primarily experimental, these systems show promise for enhancing operator performance in the anatomically distorted airways characteristic of OSA.

Continuous real-time feedback technology has been incorporated into several hybrid devices to facilitate optimal device positioning. The VivaSight^®^ (Ambu, Ballerup, Denmark) system monitors tube placement with continuous video feedback, while newer developments include pressure sensors at the blade tip of videolaryngoscopes to prevent excessive force application. Carron et al. demonstrated that this is particularly valuable in OSA patients, who often have fragile airway tissues susceptible to trauma from excessive force application [95].

Integration of these technologies with high-flow nasal oxygen (HFNO) systems has shown synergistic benefits. The combination of hybrid intubation devices with HFNO allows extended safe apnea times in OSA patients, who typically desaturate rapidly during intubation attempts. Wong et al. demonstrated that the combination of hybrid video-stylet intubation with HFNO extended safe apnea time in severe OSA patients by an average of a 2.7 min compared to conventional preoxygenation techniques [96].

While these technological advancements offer significant advantages for managing OSA airways, they are not without limitations. Cost constraints, requirements for specialized training, and potential equipment failures remain practical considerations. Additionally, many newer hybrid devices lack the extensive validation of more established techniques, particularly in emergency situations [97]. Nevertheless, the rapid evolution of these technologies continues to expand the airway management armamentarium for the challenging OSA population.

## 8. Adjunct Tools and Support Strategies

The management of difficult airways in OSA patients frequently requires adjunctive tools and support strategies to complement primary intubation devices. These adjuncts play crucial roles in enhancing success rates, reducing complications, and ensuring patient safety throughout the airway management process. Their strategic deployment, either independently or in combination with primary devices, can significantly improve outcomes in this challenging patient population.

Intubating introducers, commonly known as bougies or gum-elastic bougies (GEB), remain fundamental adjuncts in OSA airway management. Their efficacy specifically in OSA patients was evaluated by Messa et al. in a prospective cohort study involving 124 patients with severe OSA undergoing direct laryngoscopy. The authors reported that bougie-assisted intubation increased first-attempt success rates from 67% to 89% when Cormack–Lehane Grade 2b or 3 views were encountered [98]. The bougie’s small diameter and curved tip facilitate navigation through the anteriorly positioned larynx characteristic of OSA patients, even when only the posterior commissure or epiglottis is visible.

Coude-tip bougies, featuring an angled or curved distal end, offer enhanced anterior navigation particularly valuable in OSA airways. A comparative study by Shah et al. demonstrated that coude-tip bougies improved first-attempt success rates in severe OSA patients with poor laryngoscopic views compared to standard straight-tip designs (91% vs. 78%, *p* = 0.032) [99].

Articulating stylets have evolved to address the specific needs of OSA airways. The Parker Flex-It™ stylet, with its adjustable distal curvature, allows dynamic adaptation to the exaggerated anterior airway curve commonly encountered in OSA patients. Batuwitage et al. conducted a randomized controlled trial comparing the Parker Flex-It™ with standard malleable stylets in 84 OSA patients with predicted difficult airways, finding significantly higher first-attempt success rates (87% vs. 69%, *p* = 0.027) and shorter intubation times (31 s vs. 47 s, *p* = 0.004) with the articulating stylet [100]. The ability to adjust the stylet’s curvature during the intubation attempt without removing it from the field of view represents a distinct advantage in the dynamic OSA airway.

Tube exchangers serve dual purposes as both intubation adjuncts and airway exchange facilitators. In OSA patients requiring tube exchange—a particularly high-risk procedure in this population—hollow airway exchange catheters allow continuous oxygenation during the process. Mort et al. analyzed outcomes of airway exchange procedures in 42 OSA patients, reporting that use of oxygen-insufflation through hollow exchange catheters reduced desaturation incidents (SpO_2_ < 90%) from 85% to 29% compared to non-ventilated exchanges [101]. This finding has particular relevance for OSA patients who desaturate rapidly during apneic periods due to their reduced functional residual capacity and increased oxygen consumption.

Lighted stylets represent another valuable adjunct for OSA airway management. The Trachlight™ (Trachlight Technologies, Oregon, WI, USA), which provides transillumination through anterior neck tissues, offers a non-line-of-sight approach to intubation that is particularly useful when laryngoscopy proves difficult. Hung et al. compared lighted stylet intubation with direct laryngoscopy in 84 patients with OSA, finding comparable success rates but significantly fewer episodes of oxygen desaturation with the Trachlight™ (7% vs. 23%, *p* = 0.012) [102]. The reduced need for head extension and jaw manipulation with this technique helps maintain spontaneous ventilation during intubation attempts—a valuable feature in high-risk OSA patients.

Supraglottic airway devices (SADs) serve critical roles as conduits for fiberoptic-guided intubation and as rescue devices during failed intubation in OSA patients. The intubating laryngeal mask airway (iLMA^®^—Teleflex Medical, Athlone, Ireland) has shown particular utility in this context. Nickel et al. evaluated the efficacy of the iLMA in 65 OSA patients with predicted difficult airways, achieving a 92.3% success rate for blind intubation through the device and 100% success when combined with fiberoptic guidance [103]. The stabilization of pharyngeal tissues provided by the iLMA creates a protected channel for endotracheal tube passage, addressing the tissue redundancy that often complicates intubation in OSA patients.

Second-generation SADs with separation of respiratory and gastrointestinal tracts offer enhanced safety profiles for OSA patients. The ProSeal^®^ laryngeal mask (LMA) (Teleflex Medical, Athlone, Ireland) and i-gel (Intersurgical, Wokingham, UK) have demonstrated effectiveness in this population, both as primary ventilation devices and as conduits for fiberoptic intubation. Ragazzi et al. compared the i-gel with the ProSeal^®^ LMA specifically in OSA patients, finding comparable ventilation parameters but significantly faster insertion times with the i-gel (24 s vs. 42 s, *p* < 0.001) [104]. This temporal advantage may be particularly relevant in emergency situations in which OSA patients can desaturate rapidly.

High-flow nasal oxygen (HFNO) represents a breakthrough support strategy for airway management in OSA patients. By delivering up to 70 L/min of heated, humidified oxygen through nasal cannulae, HFNO extends safe apnea time during intubation attempts. Patel et al. conducted a randomized controlled trial comparing HFNO with conventional preoxygenation in 60 patients with severe OSA, demonstrating that HFNO extended the time to critical desaturation (SpO_2_ < 90%) to 750 s (*p* < 0.001) [105]. This extended safe apnea window proves particularly valuable in OSA patients who often require multiple or prolonged intubation attempts.

The combination of HFNO with videolaryngoscopy demonstrates synergistic benefits in OSA patients. Wong et al. reported that this combined approach reduced the incidence of desaturation events during intubation of severe OSA patients from 32% to 5% compared to standard preoxygenation with videolaryngoscopy. The ability to continue oxygenation during laryngoscopy and intubation attempts represents a paradigm shift in the management of these high-risk airways.

Transnasal humidified rapid insufflation ventilatory exchange (THRIVE) technique, an extension of HFNO technology, provides both oxygenation and a degree of ventilatory support during intubation attempts. Humphreys et al. evaluated THRIVE in 32 patients with severe OSA undergoing upper airway procedures, finding that continuous application throughout induction and intubation eliminated desaturation events entirely, compared to a 41% desaturation rate in historical controls using conventional techniques [106]. The combination of enhanced oxygenation with carbon dioxide clearance makes this technique particularly valuable in OSA patients with elevated metabolic demands.

Negative pressure application devices, which use suction to displace the tongue and pharyngeal soft tissues, offer a novel approach to improving visualization in OSA patients.

Potential future technologies supporting and enhancing available airway management resources may include artificial intelligence, which could both help in training and laryngoscopy performance, providing assistive support and real time laryngeal structures recognition [107,108].

While these adjuncts significantly enhance airway management capabilities in OSA patients, their optimal utilization requires thoughtful selection and integration into comprehensive management strategies. The specific combination of primary and adjunctive devices should be tailored to individual patient characteristics, available resources, and provider expertise to maximize safety and success in this challenging patient population.

## 9. Discussion

The recognition of obstructive sleep apnea as a significant risk factor for airway-related complications [109] has prompted professional societies worldwide to develop specific guidelines and algorithms addressing this patient population. These evidence-based recommendations aim to standardize approach, optimize outcomes, and reduce the significant morbidity and mortality associated with airway management in OSA patients.

The American Society of Anesthesiologists (ASA) has been at the forefront of developing comprehensive guidance for perioperative management of OSA patients. The most recent ASA Practice Guidelines, updated by Gross et al., provide a structured approach to preoperative assessment, intraoperative management, and postoperative monitoring of OSA patients [16]. These guidelines emphasize the importance of screening for undiagnosed OSA using validated tools and stratifying risk based on OSA severity, invasiveness of the procedure, and anesthetic technique. Notably, the guidelines recommend consideration of awake intubation for patients with severe OSA combined with other predictors of difficult airway management, reflecting the heightened risk in this population.

The Difficult Airway Society (DAS) of the United Kingdom has incorporated OSA-specific recommendations into their difficult airway guidelines. Frerk et al. outlined a modified approach for patients with known or suspected OSA, emphasizing thorough preoperative evaluation and preparation of alternative intubation techniques [110]. The updated DAS guidelines explicitly acknowledge the increased risk of rapid desaturation in OSA patients and recommend modified preoxygenation techniques, including 25-degree head-up positioning and continuous positive airway pressure application during preoxygenation when available. These modifications directly address the physiological limitations characteristic of OSA patients.

The Italian Society of Anesthesia, Analgesia, Resuscitation and Intensive Care (SIAARTI) has published dedicated guidelines specifically addressing airway management in patients with OSA [111]. Petrini et al. developed a comprehensive algorithm that incorporates both standard difficult airway predictors and OSA-specific factors, including severity, body mass index, and CPAP dependence [112]. A distinctive aspect of the SIAARTI approach is the recommendation for videolaryngoscopy as the primary intubation technique for all patients with moderate-to-severe OSA, reflecting the growing evidence supporting these devices in the OSA population. Unfortunately, the penetration of such guidelines has yet to be proved, with inhomogeneous application in different settings [22].

The International Airway Management society (IAMS) has contributed significantly to standardizing the approach to OSA patients. In their consensus statement, Rosenblatt et al. established a classification system for OSA severity specifically designed to guide airway management decisions [113]. This system incorporates not only the apnea-hypopnea index but also oxygen desaturation nadir, CPAP compliance, and associated comorbidities into a comprehensive risk assessment that determines the recommended intubation approach. The SAM guidelines also emphasize the importance of team preparation and communication when managing OSA patients, particularly in high-risk scenarios.

The Canadian Airway Focus Group (CAFG) has provided specific recommendations for the OSA population within their broader difficult airway management guidelines. Law et al. outlined a stepwise approach that emphasizes early identification of at-risk patients and proactive implementation of management strategies [114]. The CAFG guidelines specifically recommend continuous oximetry monitoring during all phases of airway management for OSA patients and advocate for the availability of advanced video-assisted intubation equipment whenever moderate-to-severe OSA is identified or suspected.

The European Society of Anaesthesiology (ESA) has integrated OSA considerations into their difficult airway guidelines. In the guideline by Lamperti et al., specific modifications for OSA patients include extended preoxygenation protocols, consideration of awake techniques for severe cases, and a lower threshold for utilizing advanced airway technologies [34]. The ESA guidelines also emphasize the importance of dedicated postoperative monitoring for OSA patients, recognizing the extended period of risk beyond the immediate perioperative phase. Moreover, management decisions should be individualized based on the different OSA phenotypes with upper airway difficulties.

A unique contribution to OSA airway management comes from the Society of Anesthesia and Sleep Medicine (SASM). Chung et al. developed a comprehensive perioperative management algorithm specifically for OSA patients that incorporates risk stratification, optimization strategies, and mitigation approaches [115]. Their evidence-based review suggests that patients with severe OSA should undergo intubation using either awake techniques or video-assisted devices with concurrent implementation of extended preoxygenation and apneic oxygenation protocols.

While these guidelines provide valuable frameworks, they generally acknowledge that management decisions must be individualized. Memtsoudis et al. conducted a systematic review of existing guidelines and identified significant variations in specific recommendations, highlighting the need for ongoing research and standardization [116]. However, common themes across all guidelines include the importance of early recognition, thorough preparation, and a lower threshold for utilizing advanced airway management techniques in OSA patients.

Recent updates to major guidelines have increasingly emphasized the role of technology in managing OSA airways. The newest iteration of the ASA Difficult Airway Algorithm explicitly mentions videolaryngoscopy as a primary approach for patients with OSA, reflecting the growing evidence supporting these devices in this population [117]. Similarly, the updated DAS guidelines now incorporate recommendations for high-flow nasal oxygen as a standard approach for preoxygenation and apneic oxygenation in OSA patients.

International collaborative efforts have sought to harmonize approaches across different practice environments. The World Federation of Societies of Anaesthesiologists (WFSA) has published resource-stratified guidelines for management of OSA patients that provide recommendations applicable across different healthcare settings with varying resource availability. As described by Gelb et al., these guidelines offer tiered approaches based on available equipment, staff expertise, and monitoring capabilities, ensuring that even in resource-limited settings, OSA patients can receive appropriate care [118].

While consensus exists on many aspects of OSA airway management, ongoing areas of debate and research include the optimal timing of CPAP therapy before induction, specific criteria for selecting awake versus asleep intubation techniques, and the cost-effectiveness of different advanced airway management devices in various practice settings [119]. These evolving discussions reflect the dynamic nature of this field and the commitment of professional societies to refining approaches based on emerging evidence.

## 10. Limitations

This narrative review has multiple limitations which are to be acknowledged. First, the diversity of studies investigating airway management in OSA patients render comparisons difficult. The studies included are quite heterogeneous in terms of defining OSA severity, difficult airway criteria, and endpoint. Second, most of the included studies have a small sample size and were performed under experimental conditions, and the results might not be generalized to emergency conditions or resource poor settings. The efficacy of individual devices in life-threatening situations with OSA patients is not well established. Third, there may be publication bias in the literature, with studies demonstrating favorable results with new techniques more likely to be published than those that clearly do not result in benefit, or even those that are shown to be unfavorable. This may have resulted in an overestimation of the effectiveness of new technologies. Fourth, the experience of the operator has a widely recognized impact on success with airway management techniques, in particular those with the most advanced devices. This variable is often neglected in many studies, and, for that reason, it is often hard to discriminate between the effectiveness of the device and that of the trained personnel administering it. Finally, this review largely demonstrated a technological and procedural bent toward airway management, with less attention to the broader organizational and systemic context that could impact results. Implementation strategies, need for provider training, and cost-effectiveness should all be the focus of future studies with the goal of improving airway management for OSA patients, irrespective of treatment location.

## 11. Conclusions

The management of difficult airways in patients with obstructive sleep apnea represents a significant challenge requiring a nuanced and comprehensive approach. The anatomical and physiological alterations associated with OSA create a perfect storm of intubation difficulties and heightened risk for complications. This review has highlighted the evolution of airway management strategies and technologies specifically addressing the needs of this growing patient population.

Comprehensive preoperative assessment remains the foundation of safe airway management in OSA patients, allowing adequate preparation and prudent definition of post-procedural level of care, as a paradigmatic approach to modern perioperative medicine. The integration of OSA-specific screening tools with traditional difficult airway assessment metrics enables more accurate risk stratification and appropriate selection of management strategies. This personalized evaluation must guide the selection of techniques and devices most appropriate for the individual patient.

The limitations of conventional airway management tools in the OSA population have driven remarkable technological innovation. Videolaryngoscopy has emerged as a transformative advance, significantly improving first-attempt success rates and reducing complications in OSA patients. Flexible fiberoptic intubation, particularly in the awake patient, continues to play a crucial role in high-risk scenarios, while hybrid devices combining multiple technologies offer promising new approaches to these challenging airways.

Adjunctive tools and support strategies, particularly high-flow nasal oxygen and specialized introducers, have demonstrated their value in extending safe apnea times and facilitating successful intubation in OSA patients. The strategic combination of primary and adjunctive techniques often proves more effective than reliance on a single approach.

Professional society guidelines have evolved to incorporate OSA-specific recommendations, reflecting the growing evidence base in this field. These guidelines emphasize thorough preparation, appropriate device selection, and comprehensive monitoring throughout the perioperative period.

Future directions in OSA airway management will likely include further refinement of risk prediction models, development of integrated devices combining multiple technologies, and enhanced implementation of standardized protocols. Continued research focusing specifically on the OSA population will be essential to drive evidence-based practice improvements.

The effective management of difficult airways in OSA patients ultimately requires not only appropriate technology but also thorough preparation, thoughtful strategy selection, and meticulous execution. As the prevalence of OSA continues to increase globally, the importance of optimizing airway management approaches for this challenging population will only continue to grow.

## Figures and Tables

**Table 1:** Videolaryngoscope comparison for OSA patients.

Device	Design Features	First-Attempt Success Rate	Key Advantages	Study Results
GlideScope^®^	60-degree angulated blade	91% vs. 68% with direct laryngoscopy	Superior glottic visualization in patients with high Mallampati scores and limited neck mobility	Reduced failed intubations by 76% compared to conventional approaches
McGrath^®^ MAC	Traditional Macintosh-shaped and hyperangulated blade options with CCD sensors	94% in OSA patients with predicted difficult airways	Familiar blade shape with improved visualization; reduced learning curve	Average intubation time of 38 s
C-MAC^®^ D-blade	Interchangeable blades including hyperangulated D-blade for difficult airways	Not directly specified	Superior glottic visualization (90%+ in 86% of severe OSA patients vs. 48% with direct laryngoscopy)	Effective navigation of anterior airway curvature while maintaining space for tube manipulation
Airtraq^®^	Channeled optical laryngoscope with guided tube delivery	96% vs. 72% with direct laryngoscopy	Guided tube delivery; eliminates need for separate tube manipulation	Reduced intubation time by 41% in OSA patients with Mallampati scores III–IV

## Data Availability

The original contributions presented in this study are included in the article. Further inquiries can be directed to the corresponding author.

## Abbreviations

The following abbreviations are used in this manuscript:

OSA	Obstructive Sleep Apnea
DISE	Drug-Induced Sleep Endoscopy
FI	Flexible bronchoscopy intubation
CVS	Clarus Video System
AR	Augmented reality
THRIVE	Transnasal humidified rapid insufflation ventilatory exchange
SASM	Society of Anesthesia and Sleep Medicine
WFSA	World Federation of Societies of Anaesthesiologists
ESA	European Society of Anaesthesiology
ASA	American Society of Anesthesiologists
LMAs	Laryngeal mask airways
CPAP	Continuous positive airway pressure
SIAARTI	Società Italiana di Anestesia Rianimazione e Terapia del Dolore
AHI	Apnea-Hypopnea Index
DL	Direct Laryngoscopy
VL	Videolaryngoscopy
